# Simulations of DNA-Origami Self-Assembly Reveal Design-Dependent Nucleation Barriers

**DOI:** 10.1021/acs.nanolett.2c01372

**Published:** 2022-08-29

**Authors:** Alexander Cumberworth, Daan Frenkel, Aleks Reinhardt

**Affiliations:** † AMOLF, Science Park 104, 1098 XG Amsterdam, Netherlands; ‡ Yusuf Hamied Department of Chemistry, University of Cambridge, Lensfield Road, Cambridge CB2 1EW, United Kingdom

**Keywords:** DNA origami, self-assembly, control of nucleation, isothermal assembly, coarse-grained models

## Abstract

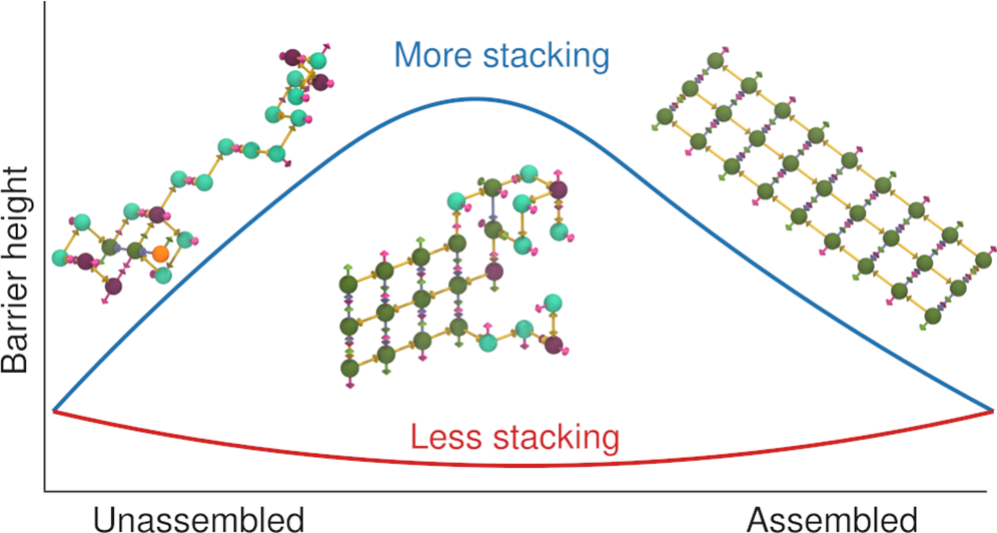

Nucleation is the rate-determining step in the kinetics of many self-assembly processes. However, the importance of nucleation in the kinetics of DNA-origami self-assembly, which involves both the binding of staple strands and the folding of the scaffold strand, is unclear. Here, using Monte Carlo simulations of a lattice model of DNA origami, we find that some, but not all, designs can have a nucleation barrier and that this barrier disappears at lower temperatures, rationalizing the success of isothermal assembly. We show that the height of the nucleation barrier depends primarily on the coaxial stacking of staples that are adjacent on the same helix, a parameter that can be modified with staple design. Creating a nucleation barrier to DNA-origami assembly could be useful in optimizing assembly times and yields, while eliminating the barrier may allow for fast molecular sensors that can assemble/disassemble without hysteresis in response to changes in the environment.

The design and production of DNA-origami structures has grown into a mature field.^1^ In these structures, a long DNA “scaffold” strand is folded into a target structure by hybridizing with a number of designed shorter “staple” strands that connect chosen binding domains on the scaffold strand. However, while there is much practical knowledge on how to optimize the assembly of DNA origamis,^2−4^ an understanding of the underlying physical mechanisms, such as the nature of any free-energy barriers to assembly and their dependence on assembly conditions, is lacking.

There is some experimental evidence that nucleation may be less important for origami self-assembly than for other assembly processes, such as crystallization. For instance, although DNA-origami assembly is often performed by slowly decreasing the temperature of a mixture with an excess of staple strands over several hours or even days,^5^ it is also possible to assemble such structures isothermally following a high-temperature denaturing step.^2,3,6−12^ Moreover, isothermal assembly has been shown to be faster for a range of designs, with the optimal temperature for this process depending on both the design of the target structure and the conditions.^2,3,7^ On the other hand, many studies on DNA origami have found hysteresis between melting and annealing as the temperature is varied,^7,13−21^ which suggests the presence of significant free-energy barriers. It has been suggested that the melting–annealing hysteresis could be attributed to a nucleation barrier to staple binding,^7,12,22^ but no numerical evidence has been given to show that such a barrier exists.

In contrast to DNA-origami assembly, nucleation has been shown to be important in the self-assembly of “DNA-brick” structures,^23,24^ which consist of a large number of short unique strands that assemble in the absence of a scaffold strand. The nucleation barrier for DNA-brick self-assembly plays an important role in allowing the error-free assembly of these many-component systems.^25^ This barrier has been studied in some depth, as control of the nucleation barrier enables the design of DNA-brick structures that have favorable assembly kinetics.^26−31^ By contrast, although DNA-origami self-assembly has been successfully modeled^16,17,32^ and subsequently validated,^33^ most existing simulation methods are too computationally expensive to allow for a systematic study of possible nucleation barriers.

However, we have previously developed a more coarse-grained model that represents DNA origami at the level of binding domains.^34^ A binding domain is the basic unit of origami design: in the final assembled state, each binding domain on the scaffold is bound to a complementary binding domain on a staple. The model accounts for hybridization free energies, coaxial stacking of helices, and steric interactions. To study nucleation behavior more accurately, we have made some modifications to the model so that it better represents stacking and steric interactions and provides a more accurate representation of the chemical potential of the staples. We provide details of the model and simulation methods in the Supporting Information (SI).

In this Letter, we use simulations with this coarse-grained model to show that nucleation can be a rate-limiting step in origami formation. In order to be able to define free-energy barriers to nucleation, we must first define order parameters that can quantify the progress of self-assembly. Here, we consider two order parameters: the numbers of (i) fully bound staples and (ii) bound-domain pairs. The former effectively accounts for the size of the cluster and is analogous to the order parameter used in classical nucleation theory and DNA-brick self-assembly, while the latter provides us with a higher-resolution view of the mechanism by which staples bind. By calculating the free energies associated with each possible value of the order parameter between assembled and unassembled states, we can determine whether barriers to assembly exist and, if so, estimate their magnitude.

To demonstrate the range of possible behaviors, we consider four systems: two that have been characterized in ref (34) (Figure 1(a) and (b)), and two other systems (see below) with as many crossovers as possible (Figure 1(c)). The two previously studied designs are (a) system S, a 24-binding-domain-scaffold system with 12 staple types, each with two binding domains (Figure 1(a)), which had been designed and simulated using the oxDNA model,^32^ and (b) system D, a 21-binding-domain-scaffold system with six two-binding-domain staple types and eight single-binding-domain staple types (Figure 1(b)), which represents a subset of the system used by Dannenberg et al.^17^ and Dunn et al.^16^ In these two systems, each binding domain has a defined sequence. We consider both sequence-specific and averaged interaction energies (details in the SI).

**Figure 1:**
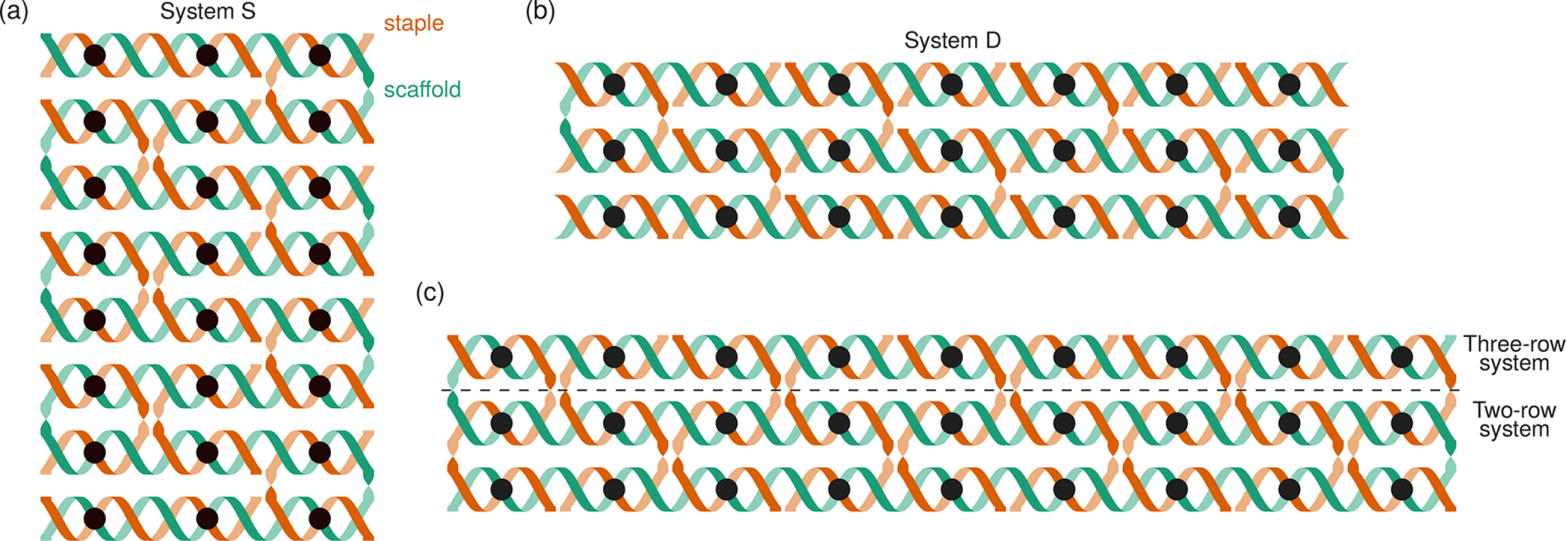
Cartoon helix representations of the systems simulated in this study. Black circles identify the binding domains, which are both the fundamental design units of DNA origami and the level to which the model is coarse-grained. (a) System S, which has a 24-binding-domain scaffold and 12 two-binding-domain staple types. (b) System D, which has a 21-binding-domain scaffold and six two-binding-domain staple types, as well as eight single-binding-domain staple types. (c) Two- and three-row systems, with a dashed line showing the cut below which is the two-row system. The two-row system has an 18-binding-domain scaffold and nine two-binding domain staple types, while the three-row system has a 27-binding-domain scaffold and nine three-binding-domain staple types.

The free energies for systems S and D show no nucleation barrier along either order parameter considered with both averaged hybridization free energies (Figures 2(a) and S8) and sequence-specific hybridization free energies (Figure S9). For computational simplicity, we define the melting temperature as the temperature at which the free energies of the fully assembled and fully unassembled states are equal. For both systems, at high (low) temperatures, the unassembled (assembled) state is favored, but at the melting temperature, the free energy as a function of the number of fully bound staples is lowest for the partially assembled state. In Figure 2(a)(i) and (ii), the free energies along the number of bound-domain pairs alternate between higher and lower values; this is consistent with the second binding domain of a staple having a lower entropic cost of binding than the first binding domain of a staple, and with a small easily surmountable barrier for staples that are near their individual melting points.

Although there is no nucleation barrier in these specific systems, it is known from experiments that hysteresis sometimes arises in DNA-origami systems. To determine conditions under which a nucleation barrier can arise, we first note that, in the context of DNA-brick self-assembly, it was shown that increasing the coordination number of the assembly units increases the barrier height.^35^ Typical DNA bricks have a coordination number of four, while for the DNA origami designs of systems S and D, it is two at most. To test whether the same principle might apply in the context of DNA origamis, we increase the number of binding domains per staple as a way of increasing the coordination number. To this end, we design a set of systems that have the maximum number of crossovers possible for a system with a given number of staple types and helices in the assembled structure (Figure 1(c)). In the assembled state of these designs, the scaffold forms a series of rows in a single plane, each of which comprises a single helix. In each column, a single staple crosses over all helices formed by the scaffold, and thus the number of binding domains per staple corresponds to the number of rows in the design. Because we are more interested in trends for these systems and because there was no qualitative difference in the results between the sequence-specific and averaged hybridization free energies for systems S and D, we consider only the averaged hybridization free energies for our designed systems. In this study, we restrict ourselves to systems that have nine binding domains per row and consider two- and three-row variants.

The free energy for the two-row system along the number of fully bound staples (Figure 2(b)(i)) at the melting temperature has no nucleation barrier. By contrast, the three-row system has a clear barrier to assembly, the maximum of which occurs nearly halfway along to the fully assembled state, at four fully bound staples, with a magnitude of ∼10*k*_B_*T* (Figure 2(b)(ii)). In both systems along the free energies of the number of bound-domain pairs, similar to the results seen for systems S and D, there are peaks at regular intervals, occurring with a frequency equal to the number of binding domains in the staple. In the three-row system, these peaks effectively add on to the barrier seen in the number of fully bound staples, giving a total barrier of around 20*k*_B_*T*. The true free-energy barriers to self-assembly are likely somewhat higher than this due to the initial binding of the first nucleotide of a domain; however, a higher-resolution model would be needed to determine their magnitudes. As the temperature is lowered to below the melting point, the barrier along the number of fully bound staples disappears after a few degrees, and the barrier along the number of bound-domain pairs also decreases substantially (Figure 3(a)). On the other hand, using averaged hybridization free energies that are 50% smaller or larger, while substantially shifting the melting temperature, has almost no effect on the barrier height (Figure S10): although they bind less (more) strongly, the entropic cost of binding is lower (higher) because of the shift in melting temperature, and the two effects appear to cancel each other.

**Figure 2:**
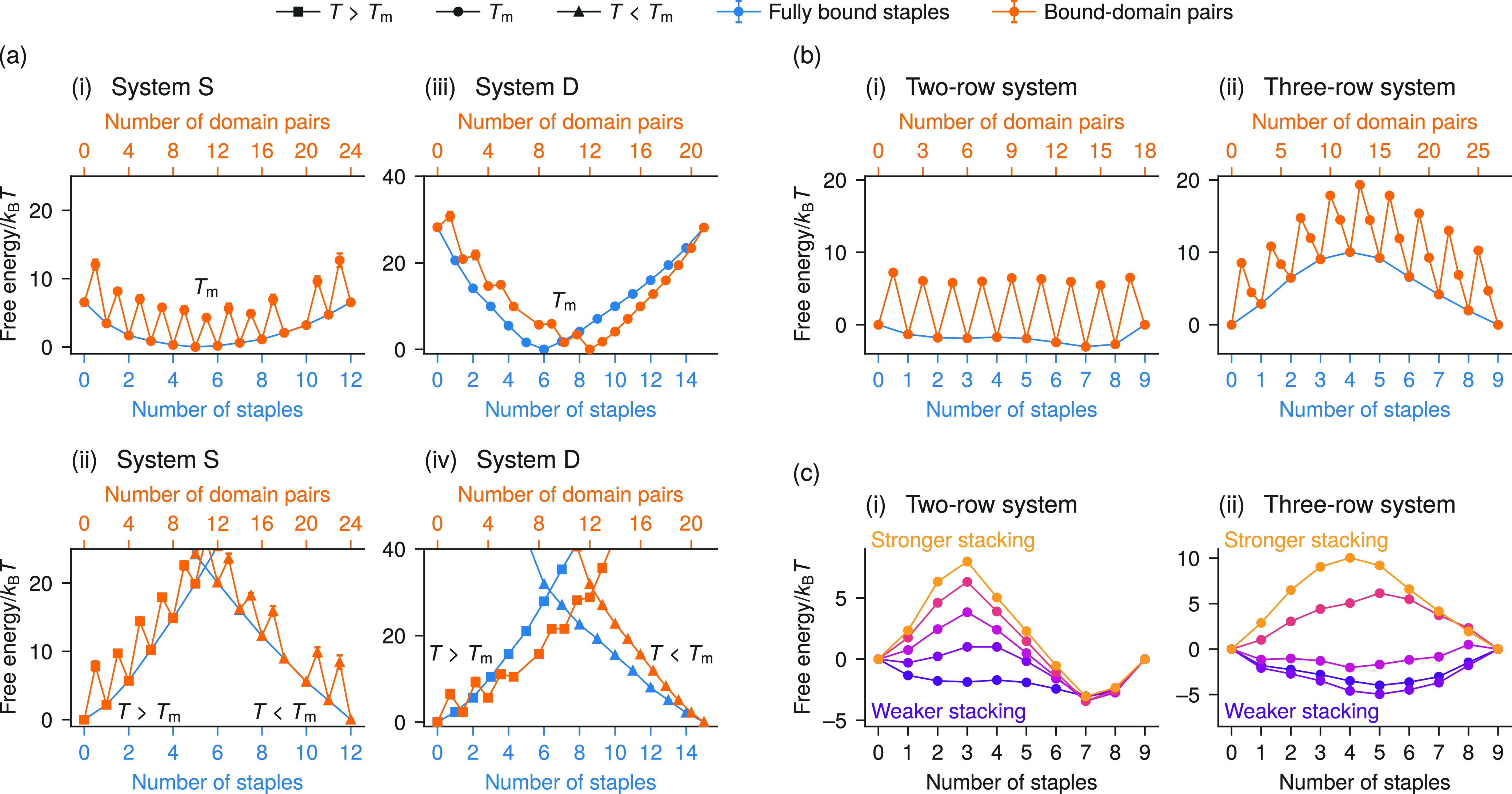
Free energies calculated for a range of values of selected order parameters. Here, both the number of fully bound staples and the number of bound-domain pairs are used as order parameters. (a) Free energies for system S, (i) and (ii), and system D, (iii) and (iv), at the melting temperature *T*_m_, (i) and (iii), and at both a temperature above and below *T*_m_, (ii) and (iv). The melting temperature is defined to be the temperature at which the free energy of the fully unassembled state is equal to the free energy of the fully assembled state. At the melting temperature for both system S (i) and system D (iii), the free energy is downhill to the favored state along the number of fully bound staples, while along the number of bound domains, only small barriers related to fully binding each staple can be seen. Below and above the melting temperatures for system S (ii) and system D (iv), the free energies are again downhill to fully assembled and unassembled states, respectively. (b) Free energies for the two-row (i) and three-row (ii) systems at the melting temperature. In the two-row system, no nucleation barrier is observed, but the three-row system shows a clear nucleation barrier along both order parameters. Free energies at several temperatures for all systems are plotted in Figure S8. (c) Free energies for the number of fully bound staples for the two-row (i) and three-row (ii) systems where the strength of the coaxial stacking parameter in the model is varied. For the two-row system, a multiplier on the stacking parameter is increased from 1 to 2 in increments of 0.25. For the three-row-system, the multiplier on the stacking parameter is decreased from 1 to 0, also in increments of 0.25. Evidently, there is a strong dependence on the coaxial stacking of not only the magnitude but even the presence of a nucleation barrier.

**Figure 3:**
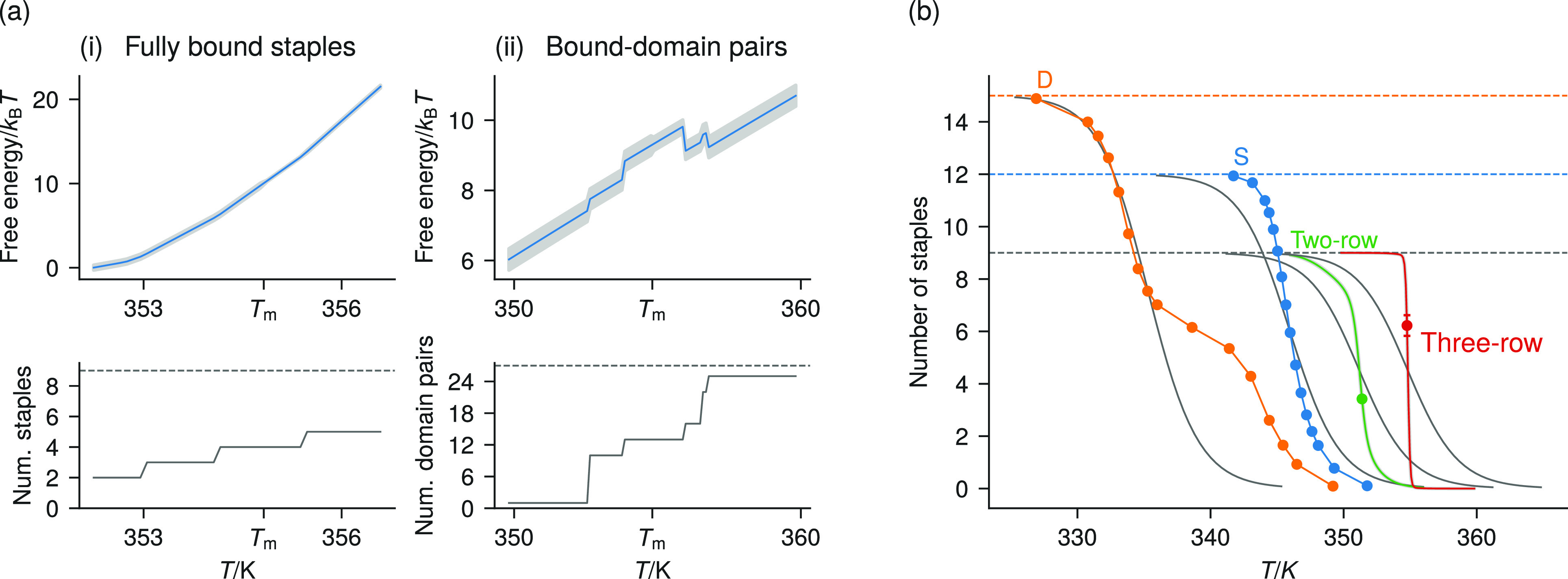
(a) Barrier height as a function of temperature for the three-row system, with the position of the peak plotted below. The plots in (i) include the entire domain over which the barrier along the number of fully bound staples is defined; outside of these temperatures, the free energies are either monotonically increasing or decreasing (Figure S8(d)). The barrier height for the number of bound-domain pairs is calculated by taking the difference between the value at the peak and the value at the local minimum; in all cases, the local minimum is located at *N*_BD_^*^ – 1, where *N*_BD_^*^ is the number of bound-domain pairs at the peak (Figures 2(a)(iv) and S8(d)). The nucleation barrier can be seen to disappear a few degrees below the melting temperature. (b) Expectation values of the number of fully bound staples as a function of the temperature. The gray lines centered on the two- and three-row system are the curves that result from assuming the binding domains act independently. (See the SI for calculation details.) The points show temperatures used in the simulations; for the two- and three-row systems, there is only one point as they are simulated with umbrella sampling. The lines between the points for system S and system D are drawn only to guide the eye, while the lines for the two- and three-row system are calculated via extrapolation. (See the SI for details.) The light gray around the extrapolated lines represents the uncertainty in the extrapolated values. In both (a) and (b), the dashed lines indicate the value of the order parameter at the fully assembled state for the system (with the corresponding color in (b)). The three-row system shows an unusually sharp transition between unassembled and assembled states.

Since all binding domains by construction have the same hybridization free energy, we might expect the systems to assemble over a relatively narrow temperature range. However, the ranges within which the S, two-row, and three-row systems transition are narrower than they would be for the same number of independent binding domains (Figure 3(b)). The three-row system displays an especially sharp transition, from entirely unbound to entirely bound in less than ∼1 K. The observed narrowing of the assembly as a function of the temperature in all studied systems implies that cooperativity is involved in the assembly process, but the nucleation barrier observed in the three-row system implies not only stronger cooperativity but also the presence of a particular type of cooperativity. By investigating the origins of this cooperativity further, we may therefore be able to determine under what conditions nucleation barriers are likely to occur in DNA-origami self-assembly.

Cooperative behavior of staples and binding domains can occur via three routes: closing of scaffold loops, initial binding of the domain of a staple to the scaffold, and coaxial stacking of binding domains adjacent in the same helix.^36^ The first route, the closing of loops, could plausibly lead to a nucleation barrier, but to be a viable pathway, it would generally require initial staples to bind more strongly than those that bind once loop closure becomes thermodynamically favorable. Since we use averaged hybridization free energies, this mechanism cannot dominate in this case. The second route leads to the jaggedness of the free energies along the number of bound-domain pairs in Figure 2(a), but it cannot explain the barrier we observe along the number of fully bound staples. We therefore focus our investigation on the stacking of adjacent binding domains along the same helix.

When a fluctuation occurs in a system so that several staples bind concurrently in such a way that they can stack with each other, the energetic gain can be sufficient to overcome the entropic cost of binding at a temperature that is higher than it would be for a given staple in isolation. The stronger the stacking per staple, whether by a more favorable stacking energy at each domain or by having more domains to stack per staple, the higher the temperature at which a cluster of staples is able to bind relative to the staples in isolation. This increased temperature difference also leads to a higher barrier, as the fluctuation needed for a given staple to bind has a higher entropic cost. We therefore anticipate that the more favorable the stacking energy, the greater the cooperativity and the larger the nucleation barrier will be.

To test this hypothesis, we run simulations where we vary the stacking energy parameter. The free energies in Figure 2(c) reveal that halving the stacking energy in the three-row system leads to the complete disappearance of the barrier. Moreover, the temperature range of the transition broadens as the stacking energy is reduced (Figure S11). On the other hand, in the two-row system, a clear barrier is seen as the stacking energy is scaled by 1.5 or more (Figure 2(c)).

We investigate the associated change in the assembly pathway by calculating expectations of individual staple states for a given number of fully bound staples. In Figure 4(a), we show that with the full stacking energy in the three-row system, after the barrier peak, there is a higher density of bound staples at the center, which becomes more intense and spreads outward as the number of fully bound staples increases. With half the original stacking energy, no such cluster appears (Figure 4(b)). A similar pattern is seen with the two-row system when comparing simulations with multipliers on the stacking energy of 0.5, 1, and 1.5 (Figure S13). These results indicate that a nucleation barrier and assembly pathway can be designed either by making the stacking energy more favorable (for example, by changing the salt concentration, by modifying the sequence pairings that occur at breakpoints, or even by using modified nucleobases which have different stacking interactions) or by increasing the number of stacking interactions in the origami design.

**Figure 4:**
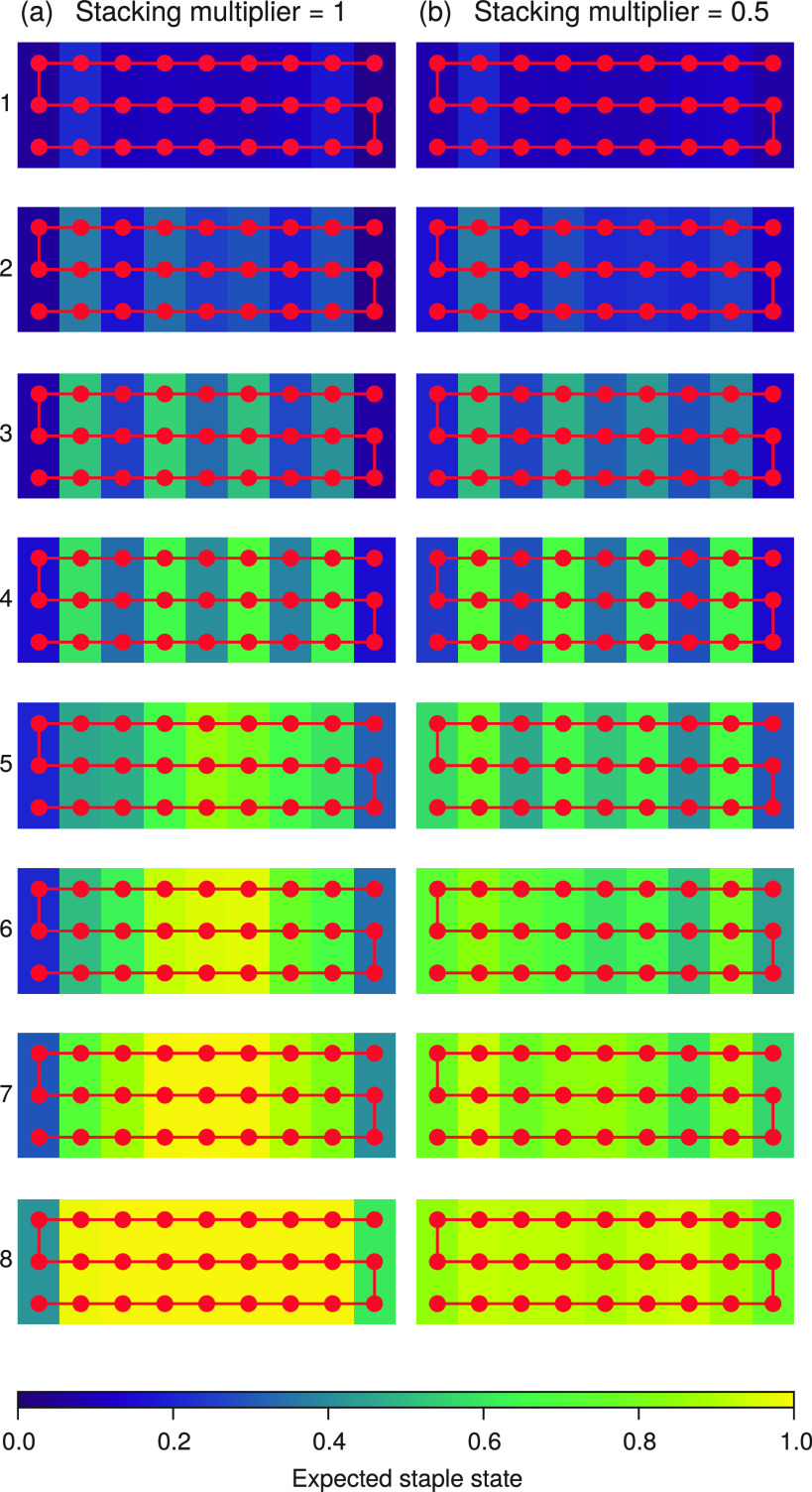
Expectation values of the staple state for each staple type at the melting temperature in the three-row system plotted as heat maps. For a given total number of fully bound staples, the heat maps show the fraction of configurations that have a staple type fully bound. The number of fully bound staples used for each set of expectation values is given to the left of the heat maps in each row. A diagram of the scaffold of the design is superimposed on each heat map. In (a), the stacking energy is set to the model’s standard value,^34^ while in (b) it is set to half that value. With full stacking, the assembly pathway indicates that nucleation tends to begin in the middle of what will become the assembled state and then grows outward; with half stacking, staples bind uniformly to the scaffold during assembly.

In summary, we have demonstrated that nucleation barriers in DNA origami depend on the coaxial stacking between helices and that some designs have no barrier at all. Small or nonexistent barriers and the consequent reversibility in the transition may be useful in a number of applications since origamis may be switched between assembled and unassembled states by changing solution conditions for functional purposes. We have also shown that origamis that do exhibit nucleation barriers can be designed by maximizing the number of crossovers in a system, thus increasing the effective coordination number, which results in a high degree of cooperativity and which in turn can be tuned by modifying the number of binding domains per staple. Since the resulting nucleation barriers are still surmountable, but the temperature range over which a transition occurs is very narrow, one can envisage applications such as molecular-scale thermometers or, by suitable functionalization, other molecular sensors.

Our results provide a rationalization for both the success of isothermal assembly and the hysteresis sometimes observed in temperature-ramp protocols: origami designs either have no barrier or have one that exists only around the melting temperature. Moreover, our results suggest that systems can be designed with barriers optimized for both assembly time and yield in an isothermal assembly protocol. If staples bind to multiple places on the scaffold concurrently, then the rearrangement times of the helices in different partially assembled chunks could be very slow, potentially leading to jammed states. A barrier allows for assembly pathways that begin locally and then grow out from that point. While the barrier observed here disappears a few degrees below the melting temperature, the assembly temperature could be tuned to be just below the melting temperature to retain the barrier and still have a good yield due to the sharp transition.

One possible difference between the self-assembly behavior of DNA origami and DNA bricks is the latter’s propensity for aggregating in such a way as to prevent full assembly. In studies of DNA bricks, it was found that at lower temperatures, incidental interactions led to the aggregation of partially assembled structures, creating a rugged free-energy landscape that inhibits the assembly process.^25,26,29,31^ Our approach cannot directly be used to simulate such aggregation in DNA-origami systems because it does not include free staples or other scaffolds; however, since the free energies along the number of bound-domain pairs are always downhill after the binding of the first domain of a staple, this would seem to imply that the staples tend to bind fully and have fewer unhybridized segments available. This makes DNA origami less prone to aggregation, as the partially assembled structures have fewer possibilities for incidental interactions with each other. This observation explains why isothermal assembly below the melting temperature can so often be successful in DNA-origami self-assembly.

Here we have focused on averaged hybridization free energies, but increased heterogeneity in the individual staple hybridization free energies could lead to lower barriers. With sufficiently disparate staple melting temperatures, the stacking energy would be insufficient to allow multiple staples to bind in such a way that they stack with each other to overcome the entropic cost of binding. If a nucleation barrier is desired, then it may prove helpful to design staple sequences that have interaction energies that are as monodisperse as possible. Similar considerations have been shown to hold for DNA bricks,^26^ although the aim in that case is usually to reduce the nucleation barrier.

In order to be able to probe the thermodynamics of DNA-origami self-assembly, we used a coarse-grained model and relatively small system sizes to ensure sufficiently rapid convergence. Although larger DNA-origami structures with complex scaffold routing might be subject to other kinds of free-energy barriers to self-assembly, many commonly used origami designs are scaled-up versions of the systems we have considered, and given that the barrier height scales with the per-staple stacking strength, rather than a global measure of the origami size, we expect our key findings to apply to such systems. Moreover, with the recent development^37−43^ of scaffolds shorter than the M13mp18 phagemid often used in origami designs, we speculate that the use of smaller scaffolds may become more popular, including scaffolds that enable highly cooperative maximum-crossover designs with monodisperse hybridization free energies.

One key message is that our results reveal that nucleation in DNA-origami self-assembly is fundamentally different from the nucleation behavior of DNA bricks and that it is possible to control, and even eliminate, the size of the barrier by judicious staple design. Such design would provide a tool for optimizing assembly times and yields and for tailoring origamis to specific functional applications.

## Notes

The data underlying this study are openly available in Zenodo at 10.5281/zenodo.6414264.

## Supporting Information Available

Full description of the coarse-grained model, simulation details, diagrams of the systems as represented with the coarse-grained model, and additional free-energy and expectation-value plots (PDF)
